# Efficacy and safety of iclepertin (BI 425809) with adjunctive computerized cognitive training in patients with schizophrenia

**DOI:** 10.1016/j.scog.2024.100340

**Published:** 2024-12-14

**Authors:** Philip D. Harvey, Sean McDonald, Eric Fu, Corey Reuteman-Fowler

**Affiliations:** a University of Miami Miller School of Medicine, 1120 NW 14th Street, 33136, Miami, FL 33136, USA; b Boehringer Ingelheim (Canada) Ltd., 5180 South Service Rd, Burlington, Ontario L7L 5Y7, Canada; c Boehringer Ingelheim Pharmaceuticals, Inc., 900 Ridgebury Road, Ridgefield, CT 06877, USA

**Keywords:** Schizophrenia, Cognitive impairment associated with schizophrenia (CIAS), Computerized cognitive training (CCT), Glycine transporter-type-1 (GlyT1) inhibitor, Iclepertin (BI 425809)

## Abstract

Despite significant patient burden, there are no approved pharmacotherapies to treat symptoms of cognitive impairment associated with schizophrenia (CIAS). This double-blind, placebo-controlled, parallel-group Phase II trial assessed the efficacy and safety of pharmacological augmentation of at-home computerized cognitive training (CCT) with iclepertin (BI 425809, a glycine transporter-1 inhibitor). Participants with schizophrenia (aged 18–50 years) on stable antipsychotic therapy, who were compliant with CCT during the run-in period, were enrolled. Patients were randomized (1:1) to once daily iclepertin 10 mg or placebo for 12 weeks, and all patients completed adjunctive CCT. At Week 12, the change from baseline in neurocognitive composite T-score of the MATRICS Consensus Cognitive Battery (primary endpoint), Schizophrenia Cognition Rating Scale interviewer total score, and Positive and Negative Syndrome Scale total score (secondary endpoints) were assessed. Performance was also assessed using Virtual Reality Functional Capacity Assessment Tool adjusted total time T-score. Of 200 randomized patients, 154 (77.0 %) completed the trial. At efficacy endpoint assessment, no differences were observed between treatment groups. Adverse events (AEs) were reported by 39 patients in the iclepertin 10 mg + CCT group and 57 patients in the placebo + CCT group; most AEs were mild to moderate. To our knowledge, this trial is the largest of its kind combining daily pharmacotherapy for CIAS with at-home CCT. Although efficacy was not demonstrated, the safety profile of iclepertin 10 mg was consistent with previous studies and no new risks were identified.

**Clinical trial registration:**

ClinicalTrials.gov identifier: NCT03859973

## 1 Introduction

Schizophrenia is a complex psychiatric disorder characterized by positive (e.g., delusions, hallucinations), negative (e.g., social withdrawal, lack of motivation), and cognitive (e.g., impairments in processing speed, working memory, and executive function) symptoms (McCutcheon et al., 2020). Cognitive impairment associated with schizophrenia (CIAS) predicts poor functional outcomes (Halverson et al., 2019), is likely a barrier to medication adherence (Acosta et al., 2012) and is associated with substantial burden on patients' daily lives (Kitchen et al., 2012). To relieve the burden on patients and reduce the financial and social burden of illness, CIAS must be adequately treated. However, there are currently no approved pharmacotherapies specifically targeting cognitive symptoms in schizophrenia (Keefe, 2019).

Positive, negative, and cognitive symptoms of schizophrenia have been linked with *N*-methyl-d-aspartate (NMDA) receptor hypofunction in the glutamatergic pathway, which is involved in synaptic plasticity and cognition (Collingridge et al., 2013; Dauvermann et al., 2017; Uno and Coyle, 2019). While studies of multiple compounds, including glutamatergic agents (i.e., D-cycloserine, cycloserine, glycine, and glycine transporter inhibitors) have suggested small beneficial effects on cognition, their clinical relevance is debatable (Sinkeviciute et al., 2018).

Computerized Cognitive Training (CCT) refers to software designed to improve cognition by promoting activity-dependent neuroplasticity practice by engaging cognitive functions (Harvey et al., 2018; Harvey and Sand, 2017). CCT may be combined with psychosocial programs in cognitive remediation therapy (Bowie et al., 2020; Harvey et al., 2018); (Sinkeviciute et al., 2018). Although only a few studies have demonstrated some beneficial effects for pharmacological interventions (Harvey and Sand, 2017; Sinkeviciute et al., 2018), evidence suggests CCT interventions may have beneficial effects on the glutamatergic system in patients with schizophrenia (Panizzutti et al., 2019). It is possible that, alongside CCT, pharmacological interventions may act synergistically to improve cognition. There has been limited testing of glutamatergic agents administered in combination with CCT (referred to as Pharmacologically Augmented Cognitive Training [PACT]) as a potential facilitating strategy (Harvey and Sand, 2017).

Iclepertin (BI 425809) is a novel, potent, and selective glycine transporter-type-1 (GlyT1) inhibitor in development for the treatment of CIAS that increases glycine levels in the synaptic cleft (Rosenbrock et al., 2023). Glycine acts as a co-agonist for NMDA receptors in excitatory glutamatergic neurotransmission (Collingridge et al., 2013; de Bartolomeis et al., 2020). Increased synaptic glycine concentration by GlyT1 inhibition may normalize NMDA receptor hypofunction in patients with schizophrenia, strengthening glutamatergic signaling and synaptic plasticity, resulting in cognitive improvement (de Bartolomeis et al., 2020). Phase I studies with iclepertin have demonstrated its safety and tolerability in healthy volunteers in single and multiple doses with a favorable pharmacokinetic profile (Moschetti et al., 2018; Rosenbrock et al., 2018; Tsuda et al., 2019). A Phase II proof-of-concept trial demonstrated improved cognition in adults with schizophrenia after 2—25 mg iclepertin treatment versus placebo at Week 12 (Fleischhacker et al., 2021).

The current Phase II trial assessed the efficacy and safety of PACT using 10 mg iclepertin combined with self-administered at-home CCT. We hypothesized that the low-level cognitive stimulation provided by the at-home CCT would augment the effects of iclepertin in treating the symptoms of CIAS in patients with schizophrenia on stable antipsychotic treatment.

## 2 Methods

### 2.1 Study design, randomization, and blinding

**Fig. 1:**
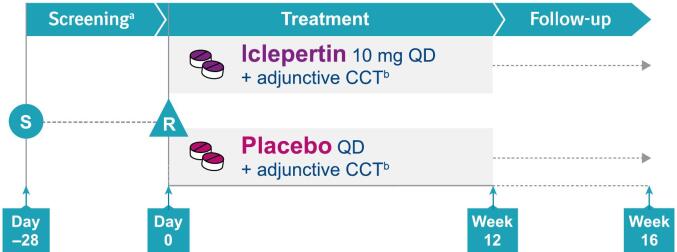
Study design. aDuring screening, patients received CCT (HappyNeuron Pro) for a 2-week run-in period, and those with sufficient adherence to CCT (completion of at least 2 h per week for 2 weeks during the run-in period) were eligible for randomization. bApproximately 30 h over 12 weeks; target of approximately 2.5 h/week. CCT, computerized cognitive training; QD, once daily; R, randomization; S, screening.

A detailed description of the study design, including full eligibility and exclusion criteria, methodology, and a complete list of endpoints has been published previously (Harvey et al., 2020). Briefly, in this Phase II, 12-week, multicenter, multinational, randomized, double-blind, placebo-controlled, parallel-group exploratory trial (NCT03859973), patients with schizophrenia across approximately 40 centers in 6 countries (USA, UK, Canada, Australia, France, New Zealand) were randomized 1:1 to receive iclepertin 10 mg or placebo for 12 weeks (Fig. 1). 10 mg was chosen as the dose level to be investigated in the present study based on the results from a proof of mechanism trial where central target engagement of iclepertin was demonstrated by a dose-dependent increase of glycine in rat and human cerebrospinal fluid (CSF). This study demonstrated a mean increase of 50 % in CSF glycine after multiple doses of 10 mg iclepertin in healthy volunteers (Rosenbrock et al., 2018).

During screening, patients received CCT (HappyNeuron Pro) for a 2-week run-in, and those with sufficient adherence to CCT (completion of at least 2 h per week for 2 weeks during the run-in period) were randomized in a blinded fashion via Interactive Response Technology at Visit 2. All randomized patients received access to an adjunctive self-administered at-home CCT for the full duration of the 12-week treatment period. Previous studies have demonstrated similar efficacy for cognitive performance with home-based self-administered training compared with office-based interventions (Biagianti et al., 2017; Wykes et al., 2011); at-home training was chosen to evaluate its feasibility in a patient population who may have difficulty accessing office-based programs (Kueider et al., 2012). Target CCT compliance was 2–3 h per week, based on the results of a large meta-analysis (Wykes et al., 2011). The minimum compliance threshold was at least 1 h per week and any less than this in a given week was recorded as a ‘CCT low compliance’ protocol deviation and triggered a follow-up from the CCT coach. Patients who completed no CCT training for three consecutive weeks or more despite follow-up from the CCT coach were classed as ‘CCT non-compliance’ protocol deviations. The trial was conducted in accordance with the Declaration of Helsinki, the International Council for Harmonization of Good Clinical Practice guidelines, applicable regulatory requirements, and BI standard operating procedures. The trial protocol and informed consent form were reviewed by the Independent Ethics Committees and/or Institutional Review Boards of the participating centers.

### 2.2 Patients

The trial included patients with schizophrenia (18–50 years of age) who were clinically stable outpatients, with no hospitalizations for worsening of schizophrenia or symptom exacerbation within 3 months before randomization. Patients could have no more than moderate positive symptoms (per the Positive and Negative Syndrome Scale [PANSS]), had to be on stable antipsychotic treatment of 1–2 antipsychotics (except for clozapine) for ≥3 months and be on their current dose for ≥30 days before randomization. All patients had to demonstrate compliance with CCT during the run-in period and have a consistent study partner to provide reliable input into the Schizophrenia Cognition Rating Scale (SCoRS) and PANSS evaluation.

### 2.3 Computerized cognitive training

The CCT program available through HappyNeuron Pro was adapted for this trial to include 22 different training tasks to train major cognitive functions often impaired in patients with schizophrenia, including processing speed, selective attention, working memory, visual memory, verbal memory, and executive functions. Task difficulty was rated from 1 to 10 for each exercise and was titrated so that progression to increasingly difficult tasks followed successful completion of previous exercises. The first time a patient completed an exercise, they began at level 1. Patients were required to successfully (≥80 % accuracy) complete this exercise twice to move up a difficulty level at their next attempt, while two consecutive failures (<60 % accuracy) would result in the patient moving down a level for their next attempt. Progression scores were generated by the HappyNeuron Pro algorithm: individual progression scores for each exercise were calculated as an average from the last two attempts of that exercise, with attempted level of difficulty adjusted for accuracy (missing attempts were scored zero). Overall progression scores were calculated as an average of all 22 individual exercise progression scores. Participants were allowed to train on their preferred tasks and training engagement was measured across the training protocol.

### 2.4 Endpoints and assessments

#### 2.4.1 Primary endpoint

The primary efficacy endpoint was the change from baseline in the Measurement and Treatment Research to Improve Cognition in Schizophrenia (MATRICS) Consensus Cognitive Battery (MCCB) neurocognitive composite T-score after 12 weeks. The MCCB comprises 10 tests assessing 7 cognitive domains, including speed of processing, attention/vigilance, working memory, verbal learning, visual learning, reasoning and problem solving, and social cognition (Nuechterlein et al., 2008). Social cognition is distinct from non-social cognition (i.e., neurocognition) (Green et al., 2019), thus the MCCB neurocognitive composite T-score was calculated without the social cognition domain. Several studies have shown that the MCCB is sensitive to CCT effects in participants with schizophrenia (Biagianti et al., 2017; Surti et al., 2023; Thomas et al., 2018).

A pre-specified subgroup analysis of the primary endpoint by cumulative CCT compliance in terms of average hours per week during treatment period (<median vs ≥ median) is also reported.

#### 2.4.2 Secondary endpoints

The key secondary endpoints were the change from baseline after 12 weeks in the SCoRS interviewer total score and the PANSS total score. The SCoRS is a 20-item interview-based assessment of cognitive deficits and the degree to which they affect day-to-day functioning (Keefe et al., 2015) that has shown preliminary evidence of sensitivity to treatment. The PANSS is a 30-item rating scale to assess the presence and severity of psychopathology (Kay et al., 1987).

#### 2.4.3 Selected exploratory endpoints

The change from baseline in the Virtual Reality Functional Capacity Assessment Tool (VRFCAT) (Keefe et al., 2016) adjusted total time T-score was an exploratory endpoint. The VRFCAT is a functional capacity measure in which participants are assessed for their ability to perform routine activities of daily living. The VRFCAT has been shown to improve with a social-cognition-focused CCT intervention in a study focused on social cognition (Nahum et al., 2021).

#### 2.4.4 Correlation between CCT engagement and cognitive improvement

In a post hoc analysis, linear regressions of change from baseline at Week 12 in MCCB neurocognitive T-score, SCoRS interviewer total score, and VRFCAT adjusted total time T-score with CCT training time were conducted. Additionally, linear regressions of the change from baseline in these cognitive measures at Week 12 with the CCT progression score by quartiles of CCT overall training time (≤11.65, >11.65 ≤ 19.38, >19.38 ≤ 29.83, >29.83) were performed.

#### 2.4.5 Safety

Safety was assessed as the percentage of patients with adverse events (AEs), serious AEs (SAEs), clinically relevant abnormalities of physical examination, vital signs, electrocardiogram, and laboratory tests. Additional safety assessments were protocol-specified AEs of special interest (AESIs), worsening of disease state (assessed by PANSS) and assessment of suicidality through electronic Columbia Suicide Severity Rating Scale (eC-SSRS).

### 2.5 Statistical analysis and sample size

A detailed description of the planned statistical analysis has been previously reported (Harvey et al., 2020). In summary, a sample size of 200 patients was considered sufficient to achieve the aims of this exploratory trial, which was not powered for any formal hypothesis testing. Demographics, baseline characteristics, and safety analyses were performed on the treated set (Harvey et al., 2020). For the primary, secondary, and VRFCAT (exploratory) endpoints, analysis was carried out on the full analysis set. The changes from baseline after 6 and 12 weeks in MCCB neurocognitive composite T-score were analyzed by a restricted maximum likelihood (REML) estimation of a mixed model for repeated measures (MMRM) adjusting for treatment by visit, baseline by visit, change from screening to baseline, and age strata. Within-patient correlation was modelled with an unstructured variance-covariance matrix. The same MMRM analysis was performed for each pre-defined subgroup with ≥25 patients. For SCoRS and VRFCAT endpoints, an analysis of covariance was performed, whereas REML estimation of a MMRM was performed for PANSS; these analyses were adjusted for age strata and the corresponding baseline value (Harvey et al., 2020). Post hoc exploratory regression analysis of associations between the cognitive measures, CCT training time, and CCT progression score were only performed in patients receiving placebo.

## 3 Results

### 3.1 Patient disposition and demographics

**Fig. 2:**
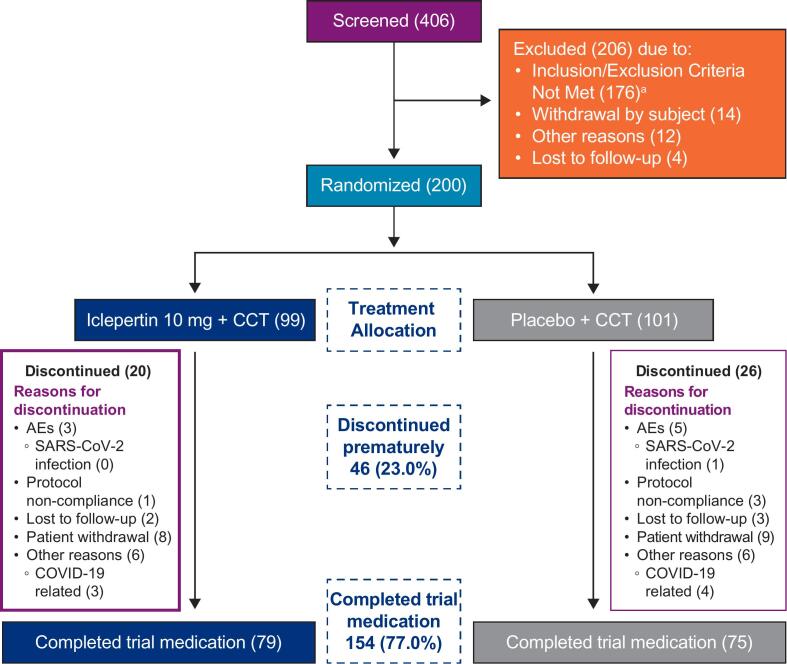
Patient disposition. ^a^Of the 176 patients who did not meet the inclusion/exclusion criteria, 47 patients were unable to properly use the CCT device or were non-compliant with the requirements of the at-home CCT during the 2-week run-in period. AEs, adverse events; CCT, computerized cognitive training; COVID-19, coronavirus disease 2019; SARS-CoV-2, severe acute respiratory syndrome coronavirus 2.

Of 406 screened patients, 200 were randomized (iclepertin group: iclepertin 10 mg + CCT [*n* = 99]; placebo group: placebo + CCT [*n* = 101]) and 154 (77.0 %) completed the trial medication. A total of 46 patients (23.0 %) prematurely discontinued treatment; the discontinuation rate was similar across groups (iclepertin group [*n* = 20]; placebo group [*n* = 26]; Fig. 2). Most patients were randomized in the USA (148/200 [74.0 %]), 136 (68.0 %) were male, and 98 (49.0 %) were white. The VRFCAT scores were relatively high at baseline for both treatment groups (Table 1). There was no substantial difference in alcohol and nicotine use between treatment groups. The mean exposure to trial medication and CCT was comparable for both treatments (Supplementary Table 1).

### 3.2 Efficacy

#### 3.2.1 Primary endpoint

The adjusted mean (standard error [SE]) change from baseline in MCCB neurocognitive composite T-score after 12 weeks was 1.6 (0.6) points for iclepertin and 2.5 (0.6) points for placebo. The MMRM analysis did not show a treatment difference between iclepertin 10 mg + CCT and placebo + CCT groups (adjusted mean difference [SE] versus placebo, −0.9 [0.9], *p* = 0.3101; Table 2). However, the adjusted mean [SE] change from baseline in the MCCB neurocognitive composite T-score at Week 12 indicated a slight improvement for the iclepertin 10 mg + CCT group, albeit not reaching statistical significance (Table 2).

**Table 1:** Patient demographics and baseline assessments.

Baseline Characteristic	Placebo + CCT (*n* = 101)	Iclepertin 10 mg + CCT (*n* = 99)	Total (*N* = 200)
**Demographics**			
Male, n (%)	71 (70.3)	65 (65.7)	136 (68.0)
Female, n (%)	30 (29.7)	34 (34.3)	64 (32.0)
Race, n (%)			
White	51 (50.5)	47 (47.5)	98 (49.0)
Black or African American	40 (39.6)	44 (44.4)	84 (42.0)
Asian	5 (5.0)	4 (4.0)	9 (4.5)
Other⁎	5 (5.0)	2 (2.0)	7 (3.5)
Age (years), mean (SD)	38.5 (7.7)	37.9 (8.1)	38.2 (7.9)
Time since diagnosis of schizophrenia (years), mean (SD)	13.9 (8.4)	13.1 (8.4)	13.5 (8.4)

**Baseline assessments**			
MCCB neurocognitive composite T-score, mean (SD)	34.0 (11.3)	33.5 (12.0)	33.7 (11.6)
SCoRS interviewer total score, mean (SD)†	34.8 (8.9)	36.3 (8.6)	35.5 (8.7)
PANSS total score, mean (SD)	63.3 (14.5)	65.8 (14.5)	64.5 (14.5)
VRFCAT summary score, mean (SD)	44.9 (15.2)	45.1 (13.9)	45.0 (14.6)

**Antipsychotic concomitant therapies**			
Patients with 1 antipsychotic, n (%)	79 (78.2)	78 (78.8)	157 (78.5)
Patients with 2 antipsychotics, n (%)	20 (19.8)	20 (20.2)	40 (20.0)
Patients with oral antipsychotics, n (%)	78 (77.2)	75 (75.8)	153 (76.5)

CCT, computerized cognitive training; MCCB, MATRICS Consensus Cognitive Battery; PANSS, Positive and Negative Syndrome Scale; SCoRS, Schizophrenia Cognition Rating Scale; SD, standard deviation; VRFCAT, Virtual Reality Functional Capacity Assessment Tool.

⁎ Includes American Indian or Alaska Native, Native Hawaiian or Other Pacific Islander, or those in multiple racial categories.

† Baseline was available for 94 and 91 patients in the placebo + CCT arm and iclepertin + CCT arm, respectively.

**Table 2:** Change from baseline in MCCB neurocognitive composite T-score by treatment groups at Weeks 6 and 12 (FAS).

	Week 6		Week 12 (EoT)	
	Placebo + CCT (*n* = 86)	Iclepertin 10 mg + CCT (*n* = 88)	Placebo + CCT (*n* = 86)	Iclepertin 10 mg + CCT (*n* = 88)
**Change from baseline**				
Adjusted mean (SE)	1.8 (0.5)	1.8 (0.5)	2.5 (0.6)	1.6 (0.6)
95 % CI	(0.8, 2.7)	(0.9, 2.8)	(1.2, 3.7)	(0.4, 2.8)

**Iclepertin vs placebo**				
Adjusted mean difference (SE)	0.1 (0.7)		−0.9 (0.9)	
95 % CI	(−1.3, 1.4)		(−2.6, 0.8)	
*p*-value	0.9099		0.3101	

Data was analyzed by a MMRM model using an unstructured within-subject variance-covariance matrix and included the following fixed effects: categorical factors of planned treatment, visit, planned treatment by visit interaction, and age group as well as continuous covariates of baseline value, baseline by visit interaction, and change from screening to baseline value. Increases in MCCB scores indicate improvements.

CCT, computerized cognitive training; CI, confidence interval; EoT, end of treatment; FAS, full analysis set; MCCB, MATRICS Consensus Cognitive Battery; MMRM, mixed models for repeated measures; SE, standard error.

**Fig. 3:**
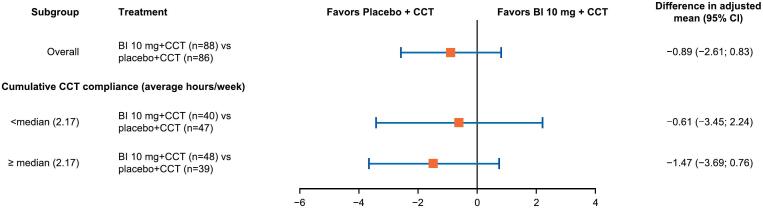
Treatment effects versus placebo at Week 12 for MCCB neurocognitive composite T-score overall, and by cumulative CCT compliance (FAS). The adjusted mean difference between iclepertin and placebo groups was obtained by a REML estimation of a MMRM model with unstructured variance-covariance matrix fitted to each subgroup level. CCT, computerized cognitive training; CI, confidence interval; FAS, full analysis set; MCCB, MATRICS Consensus Cognitive Battery; MMRM, mixed model for repeated measures; REML, restricted maximum likelihood.

While the subgroup analysis by CCT training adherence was generally consistent with the overall analysis, a slight trend towards a higher improvement in the placebo arm was observed for the subgroup of patients with cumulative CCT compliance ≥ median (2.17 h per week; Fig. 3).

#### 3.2.2 Secondary endpoints and selected exploratory endpoints

There was no treatment difference between iclepertin and placebo groups in the SCoRS interviewer total score (adjusted mean difference [SE] versus placebo, 0.6 [0.9], *p* = 0.5237) or PANSS total score (−0.7 [1.4], *p* = 0.6420) at Week 12 (Table 3 **A and 3B**). Similar to the primary endpoint, the adjusted mean [SE] change from baseline in the SCoRS interviewer total score at Week 12 indicated a slight improvement (not statistically significant) for the iclepertin 10 mg + CCT group (Table 3 **A**).

**Table 3:** Change from baseline in (A) SCoRS interviewer total score at Week 12 (FAS), (B) PANSS total score at Week 6 and Week 12 (FAS), and (C) VRFCAT total time T-score at Week 12 (FAS).

A. SCoRS		
	Placebo + CCT (*n* = 75)	Iclepertin 10 mg + CCT (*n* = 82)
**Change from baseline at Week 12 (EoT)**		
Adjusted mean (SE)	−3.1 (0.7)	−2.5 (0.6)
95 % CI	(−4.4, −1.8)	(−3.7, −1.3)

**Iclepertin vs placebo**		
Adjusted mean difference (SE)	0.6 (0.9)	
95 % CI	(−1.2, 2.4)	
p-value	0.5237	


B. PANSS				
	Week 6		Week 12 (EoT)	
	Placebo + CCT (n = 85)	Iclepertin 10 mg + CCT (n = 88)	Placebo + CCT (*n* = 85)	Iclepertin 10 mg + CCT (n = 88)
**Change from baseline**				
Adjusted mean (SE)	−2.0 (1.0)	−0.6 (0.9)	−2.1 (1.0)	−2.7 (1.0)
95 % CI	(−3.9, −0.1)	(−2.5, 1.2)	(−4.1, −0.1)	(−4.7, −0.8)

**Iclepertin vs placebo**				
Adjusted mean difference (SE)	1.3 (1.3)		−0.7 (1.4)	
95 % CI	(−1.3, 4.0)		(−3.5, 2.2)	
p-value	0.3194		0.6420	


C. VRFCAT		
	Placebo + CCT (*n* = 65)	Iclepertin 10 mg + CCT (*n* = 62)
**Change from baseline at Week 12 (EoT)**		
Adjusted mean	−2.6	−3.4
95 % CI	(−5.0, −0.2)	(−5.8, −1.0)

**Iclepertin vs placebo**		
Adjusted mean difference (SE)	−0.8 (1.7)	
95 % CI	(−4.2, 2.6)	
p-value	0.6542	

Data was analyzed using the ANCOVA model which included the following fixed effects: categorical factors of planned treatment and age group as well as a continuous covariate of baseline value. Decreases in SCoRS rating indicates improvements.

Data was analyzed by a MMRM model using an unstructured within-subject variance-covariance matrix and included the following fixed effects: categorical factors of planned treatment, visit, planned treatment by visit interaction, and age group as well as a continuous covariates of baseline value and baseline by visit interaction. Decreases in PANSS scores indicate improvements.

Data was analyzed by an ANCOVA model which included the following fixed effects: categorical factors of planned treatment and age group as well as a continuous covariate of baseline value. Decreases in VRFCAT summary scores indicate worsening performance.

ANCOVA, analysis of covariance; CCT, computerized cognitive training; CI, confidence interval; EoT, end of treatment; FAS, full analysis set; MMRM, mixed models for repeated measures; PANSS, Positive and Negative Syndrome Scale; SCoRS, Schizophrenia Cognition Rating Scale; SD, standard deviation; SE, standard error; VRFCAT, Virtual Reality Functional Capacity Assessment Tool.

There was also no treatment difference between iclepertin and placebo groups in the VRFCAT adjusted total time T-score (adjusted mean difference [SE] versus placebo, −0.8 [1.7], *p* = 0.6542; Table 3**C**). However, the adjusted mean [SE] change from baseline in the VRFCAT adjusted total time T-score at Week 12 indicated a slight deterioration for the iclepertin 10 mg + CCT group (Table 3**C**).

#### 3.2.3 Correlation between CCT training time and cognitive improvement

**Fig. 4:**
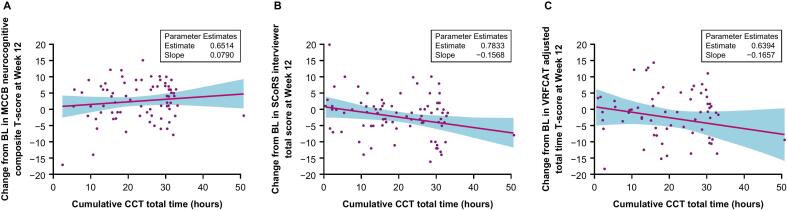
CCT Training time versus change from baseline at Week 12 in (A) MCCB neurocognitive composite T-score, (B) SCoRS interviewer total score, and (C) VRFCAT adjusted total time T-score. CCT, computerized cognitive training; MCCB, MATRICS Consensus Cognitive Battery; PANSS, Positive and Negative Syndrome Scale; SCoRS, Schizophrenia Cognition Rating Scale; VRFCAT, Virtual Reality Functional Capacity Assessment Tool. The CCT progression score of an attempt is based on the level attempted adjusting for accuracy: level×10 for 80 % or higher accuracy, (level – 0.5) × 10 for 60 % to < 80 % accuracy, (level – 1.5) × 10 for < 60 % accuracy, and 0 for no attempts, for exercise levels that progress from 1 to 10.

In placebo patients, there was no correlation between CCT overall training time and the change from baseline at Week 12 in MCCB neurocognitive composite T-score (estimated slope, SE: 0.08 [0.05] per hour) or VRFCAT adjusted total time T-score (−0.17 [0.12] per hour), while the data suggest a negative correlation between CCT overall training time and changes from baseline at Week 12 in SCoRS interviewer total score (−0.16 [0.07]; Figs. 4A–C).

The linear regression of change from baseline in cognitive measures at Week 12 on CCT progression score was also analyzed by subgroups of CCT overall training time quartiles. Generally, no pattern of correlation is suggested by the data, except a positive correlation between the CCT progression score and the MCCB neurocognitive composite T-score at Week 12 in patients with >19.38 to 29.83 h of overall CCT training, and a negative correlation between the CCT progression score and the SCoRS interviewer total score at Week 12 in patients with ≤11.65 h of overall CCT training.

### 3.3 Safety

Overall, 39/99 (39 %) patients in the iclepertin group and 57/101 (56 %) patients in the placebo group reported ≥1 AE, and most AEs were mild to moderate (Table 4). Between treatment groups, similar proportions of AEs led to discontinuation of trial medication. There were very few severe AEs and SAEs, and no AESIs or deaths.

**Table 4:** Overall summary of adverse events (treated set).

Category of AE, N (%)	Iclepertin 10 mg + CCT (n = 99)	Placebo + CCT (n = 101)
Any AE	39 (39.4)	57 (56.4)
Severe AEs	1 (1.0)	3 (3.0)
Investigator-defined drug-related AEs	17 (17.2)	21 (20.8)
AEs leading to discontinuation of trial medication	3 (3.0)	4 (4.0)
AESIs	0	0
SAEs^a^	1 (1.0)	2 (2.0)
Fatal	0	0
Life-threatening	0	0
Persisting or significant disability/incapacity	0	0
Requiring or prolonging hospitalization	1 (1.0)	1 (1.0)
Congenital anomaly or birth defect	0	0
Other	0	1 (1.0)

An adverse event was defined as any untoward medical occurrence. An SAE was any AE which fulfilled one of the following criteria: resulted in death, was life-threatening, required inpatient hospitalization, required prolonging of existing hospitalization, resulted in a persistent/significant disability/incapacity, or deemed serious based on appropriate medical judgment.

AE, adverse events; AESI, adverse events of special interest; CCT, computerized cognitive training; SAE, serious adverse events.

a A patient could be counted in >1 seriousness criterion.

Overall, no clinically relevant changes in laboratory parameters and vital signs were observed. A similar number of patients in both groups (iclepertin [*n* = 8]; placebo [*n* = 7]) had disease worsening indicated by the PANSS positive score ≥ 5 and 4 patients in each treatment group with PANSS total score ≥ 15. In the iclepertin and placebo groups, there were 7 and 8 patients, respectively, with ≥1 event of suicidal ideation during treatment. No events of C-SSRS type 4 or 5, suicidal behavior, or self-injury were reported.

## 4 Discussion

Results from this Phase II study to assess the efficacy and safety of iclepertin versus placebo for the treatment of CIAS demonstrated that iclepertin 10 mg had a favorable safety profile and was well tolerated. Consistent with previous Phase I and II trials, the iclepertin group had lower incidence of AEs versus placebo (39 % versus 56 %), and headache was the most common AE (Fleischhacker et al., 2021; Moschetti et al., 2018; Rosenbrock et al., 2018; Tsuda et al., 2019). However, the primary and secondary efficacy endpoints were not met in this trial. After 12 weeks of treatment, no difference was observed between the iclepertin and placebo groups in the MCCB neurocognitive composite T-score, SCoRS interviewer total score, or PANSS total score. Further, results from the exploratory VRFCAT endpoint also indicated no improvements in functional capacity.

Although the present study failed to show positive efficacy results, 3 Phase III trials are ongoing to examine the efficacy, safety, and tolerability of iclepertin in improving cognition and functioning in a larger group of patients, over a longer treatment period (26 weeks); however, these trials do not include an adjunctive CCT: CONNEX-1, CONNEX-2, and CONNEX-3 (NCT04846868, NCT04846881, NCT04860830).

It is unclear whether the failure to meet efficacy endpoints in the current study truly indicates that iclepertin does not affect CIAS in its own right, or if there are methodological considerations which may have contributed to the lack of positive results. In the previous Phase II proof-of-concept trial that demonstrated improved cognition in adults with schizophrenia after 2—25 mg iclepertin (Fleischhacker et al., 2021) the effect size seen was significant, but numerically small. It is possible that in the current study the sample size was too small and baseline VRFCAT scores too high to detect iclepertin monotherapy effects. Results from the larger, ongoing Phase III study are expected soon and should help answer this question.

Similarly, it is difficult to say whether the results of the current study indicate iclepertin has no augmenting effect on CCT. Use of CCT to improve cognition is a relatively recent development, and there are minimal comparative data regarding efficacy of specific CCT programs. Although similar studies have demonstrated comparable efficacy of home-based and office-based interventions (Biagianti et al., 2017; Wykes et al., 2011), it is possible that providing an office-based training environment and observation by healthcare professionals could have a slightly higher impact on training engagement and subsequent improvement in outcomes. Most previous studies using the HappyNeuron Pro CCT program deployed the training system as part of a comprehensive rehabilitation program, which includes training in areas such as strategic monitoring, bridging, and functional adaptation skills in order to achieve large and durable effects on functional competence and real-world functioning (Bowie et al., 2012). In contrast, the intention of using CCT in the present study was not to effect cognitive change per se, but rather to provide a low level of cognitive stimulation to augment the effects of pharmacological treatment, in the most standardized possible way. Further to this, providing CCT in the home setting allowed us to evaluate the feasibility of at-home training in this patient population who may have had difficulty accessing traditional training programs (Kueider et al., 2012). These differences mean a direct efficacy and mechanism of action comparison to the use in the current study is not possible (HappyNeuron-Pro., 2012). In two recent meta-analyses of cognitive training for the treatment of psychosis, at-home HappyNeuron Pro was not used in any of the cited articles (Penney et al., 2022; Vita et al., 2021). The lack of available efficacy data for HappyNeuron Pro alone makes the present results difficult to interpret, and it is difficult to speculate as to whether larger effects may have been seen from using a different CCT program. In the subgroup analysis of change from baseline in the MCCB neurocognitive composite score, a slight tendency towards greater improvement in the placebo + CCT group was observed for the subgroup of patients with total CCT compliance ≥ median 2.17 h/week. However, there was no correlation between CCT training time and cognitive outcomes. These mixed findings suggest no strong association between CCT and efficacy outcomes and add to the difficulty of differentiating treatment effects from CCT effects.

Another factor that complicated the interpretation of the trial results was the absence of a non-CCT placebo arm. A recent publication reported results from a similar trial to the current study, wherein the impact of a GlyT1 inhibitor (PF-03463275) was investigated in combination with computer-based cognitive training (HappyNeuron Pro) to treat CIAS in a cross-over trial with no non-CCT placebo arm, and found no augmentation of the effects of cognitive training (Surti et al., 2023). One possible explanation offered by the authors is that it may be difficult to interpret the augmenting effects of two interventions in the context of one another, and an additional non-CCT placebo arm may help to produce clearer results (Surti et al., 2023), a consideration which may also be relevant in the present study. In contrast, a three-arm study comparing computerized cognitive training alone, in-person, group delivered functional adaptation skills training alone, or a combination of both (which met the criteria for being designated as “cognitive remediation”) and had no inactive placebo control arm, found statistically significant cognitive gains in both CCT and combined treatment arms, as well as gains in everyday functioning that were significant in the combined treatment arm (Bowie et al., 2012).

A potential limitation of this study is the high scores on VRFCAT assessment at baseline. The VRFCAT total time T-scores of ∼45 were observed in both treatment groups in this study; while average VRFCAT total time T-scores of 49.7 and 32.5 have previously been obtained from healthy volunteers without cognitive impairment, and patients with schizophrenia, respectively (Keefe et al., 2016). While these may appear to be in contrast with the MCCB baseline scores of ∼34.0 (both treatment groups), which were in the expected, impaired range, it is important to remember that the MCCB is a paper and pencil test, while the VRFCAT is completed digitally. It is plausible that participants with advanced technological skills may have been more inclined to volunteer for a computer training study, meaning the technological performance of the population may have outstripped their general cognitive ability. This could partly explain the lack of efficacy observed in the VRFCAT endpoint and suggests a potential selection bias of patients with a high level of functional capacity. Another limitation of this study is that the trial site selection was restricted, given that the CCT program was only available in English and French languages. Finally, the study was exploratory in nature, which may affect its interpretation, and not powered to detect differences between the treatment groups beyond the primary measure.

Despite the lack of positive efficacy results, the present study had several strengths. Foremost, this was the largest trial of its kind to combine CCT and pharmacotherapy for schizophrenia in 200 patients with CIAS. The recent publication by Surti TS et al. examined the effects of a GlyT1 inhibitor (PF-03463275) in combination with CCT in a smaller sample (71 patients randomized) (Surti et al., 2023). The successful implementation of the at-home CCT component represents an additional strength of the current study; on average, patients completed approximately 21.3 h of CCT in the iclepertin group and 19.4 h in the placebo group over a duration of 12 weeks. The adherence results presented here compare favorably to previous home-based training interventions (Biagianti et al., 2017; Loewy et al., 2022; Surti et al., 2023).

## 5 Conclusions

To our knowledge, this trial is the largest of its kind combining daily pharmacotherapy for CIAS with at-home CCT. Even though efficacy could not be demonstrated, the safety results of iclepertin 10 mg were consistent with previous clinical studies and no new risks were identified. Three multinational Phase III trials (CONNEX-1, −2, and − 3) are ongoing to further investigate the efficacy and safety of iclepertin in improving cognition and daily functioning (without utilizing an adjunctive CCT) in patients with schizophrenia.

The following is the supplementary data related to this article.

Supplementary Table 1 Exposure to trial medication and CCT–TS. Supplementary Table 1

## CRediT authorship contribution statement

**Philip D. Harvey:** Writing – review & editing, Writing – original draft, Supervision, Methodology, Investigation, Conceptualization. **Sean McDonald:** Writing – review & editing, Writing – original draft, Supervision, Project administration, Methodology, Formal analysis, Data curation, Conceptualization. **Eric Fu:** Writing – review & editing, Writing – original draft, Validation, Methodology, Formal analysis, Data curation. **Corey Reuteman-Fowler:** Writing – review & editing, Writing – original draft, Supervision, Project administration, Methodology, Conceptualization.

## Author contributions

SMD and PH were involved in the conception and design of the study. SMD and EF collected the data. CRF and PH were involved in data analysis, interpretation, and dissemination. All authors were involved in the preparation and review of the manuscript and approved the final version submitted for publication.

## Authors' statement

This paper received editorial assistance funded by Boehringer-Ingelheim.

The authors of the paper acknowledge that they are solely responsible for the article content.

## Funding

This study was funded by Boehringer Ingelheim International GmbH (ClinicalTrials.gov identifier: NCT03859973; BI study number: 1346–0038).

## Declaration of competing interest

**CRF, SM,** and **EF** are employees of Boehringer Ingelheim. **PDH** has received consulting fees or travel reimbursements from Alkermes, Boehringer Ingelheim, Karuna Therapeutics, Minerva Neuroscience, and Sunovion/DSP Pharma during the past year. He is Chief Science Officer of i-Function, Inc. (whose products were not used in this trial) and receives royalties from WCG Endpoint Solutions (owner of the VRFCAT) for the Brief assessment of Cognition. PDH does not receive royalties for the sale or use of VRFCAT.

## Data Availability

To ensure independent interpretation of clinical study results and enable authors to fulfil their role and obligations under the ICMJE criteria, Boehringer Ingelheim grants all external authors access to relevant clinical study data. In adherence with the Boehringer Ingelheim Policy on Transparency and Publication of Clinical Study Data, scientific and medical researchers can request access to clinical study data, typically, one year after the approval has been granted by major Regulatory Authorities or after termination of the development program. Researchers should use the https://vivli.org/ link to request access to study data and visit https://www.mystudywindow.com/msw/datasharing for further information.
