# Supplementary material for: Efficacy and safety of iclepertin (BI 425809) with adjunctive computerized cognitive training in patients with schizophrenia
Source: Schizophr Res Cogn. 2024 Dec 14;40:100340. doi: 10.1016/j.scog.2024.100340 (PMC11699300; doi:10.1016/j.scog.2024.100340)
Supplement: Supplementary Table 1 — Exposure to trial medication and CCT–TS. [file mmc1.docx]

### **Supplementary Table 1** Exposure to trial medication and CCT–TS.

|  | **Iclepertin 10 mg + CCT**  **(n=99)** | **Placebo + CCT**  **(n=101)** |
| --- | --- | --- |
| Treatment time (days) | | |
| Mean (SD) | 74.7 (21.7) | 72.2 (25.2) |
| Median (min, max) | 84.0 (1, 95) | 84.0 (1, 106) |
| Treatment time categories, n (%) |  |  |
| <30 days | 8 (8.1) | 14 (13.9) |
| 30 to <42 days | 2 (2.0) | 1 (1.0) |
| 42 to <60 days | 8 (8.1) | 4 (4.0) |
| 60 to <90 days | 78 (78.8) | 79 (78.2) |
| ≥90 days | 3 (3.0) | 3 (3.0) |
| CCT total time spent (h) |  |  |
| Mean (SD) | 21.3 (10.4) | 19.4 (10.7) |
| Median (min, max) | 23.4 (0, 46.6) | 19.4 (0, 51.0) |

CCT, computerized cognitive training; SD, standard deviation; TS, treated set.
